# 829. 5-Flucytosine Longitudinal Antifungal Susceptibility Testing of *Cryptococcus neoformans*: A Sub-study of the EnACT Trial Testing Oral Amphotericin

**DOI:** 10.1093/ofid/ofad500.874

**Published:** 2023-11-27

**Authors:** Thomas McHale, Andrew Akampurira, Elliot Gerlach, Mucunguzi Atukunda, Melanie R Nicol, Darlisha A Williams, David Meya, David R Boulware

**Affiliations:** University of Minnesota, Cambridge, Nebraska; Infectious Diseases Institute, Makerere University, 256, Kampala, Uganda; University of Minnesota, Cambridge, Nebraska; Infectious Diseases Institute, Makerere University, 256, Kampala, Uganda; University of Minnesota, Cambridge, Nebraska; University of Minnesota, Cambridge, Nebraska; Infectious Diseases Institute, Makerere University, 256, Kampala, Uganda; University of Minnesota, Cambridge, Nebraska

## Abstract

**Background:**

The EnACT trial was a phase 2 randomized clinical trial conducted in Uganda, which evaluated a novel orally delivered lipid nanocrystal (LNC) amphotericin B for treatment of cryptococcal meningitis. Oral LNC amphotericin B applies nanotechnology, which hopes to improve intracellular drug delivery to affected tissues while reducing extracellular concentrations and thereby adverse events. When flucytosine (5-FC) is used as monotherapy, it can induce stable, highly resistant mutants of *Cryptococcus*. Since LNC amphotericin is a novel drug delivery mechanism, we assessed whether resistance to 5-FC develops in this context.

Results from the ACTA and AMBITION Clinical Trials

**Figure:**
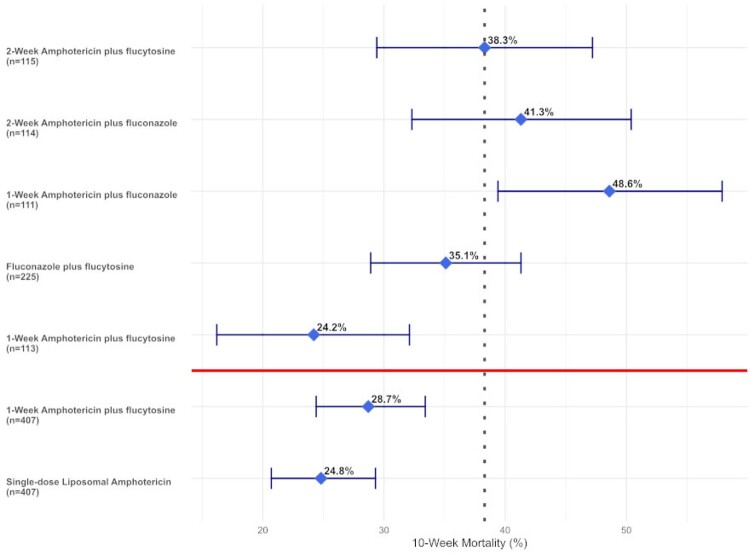

10-week mortality by antifungal regimen for cryptococcal meningitis from the clinical trial by Molloy et al. (ACTA) in top section and Jarvis et al. (AMBITION) in bottom section. Mean survival for each group is represented by diamonds and 95% confidence interval represented by error bars.

Encapsulation of Amphotericin B in a Lipid nanocrystal Particle

**Figure:**
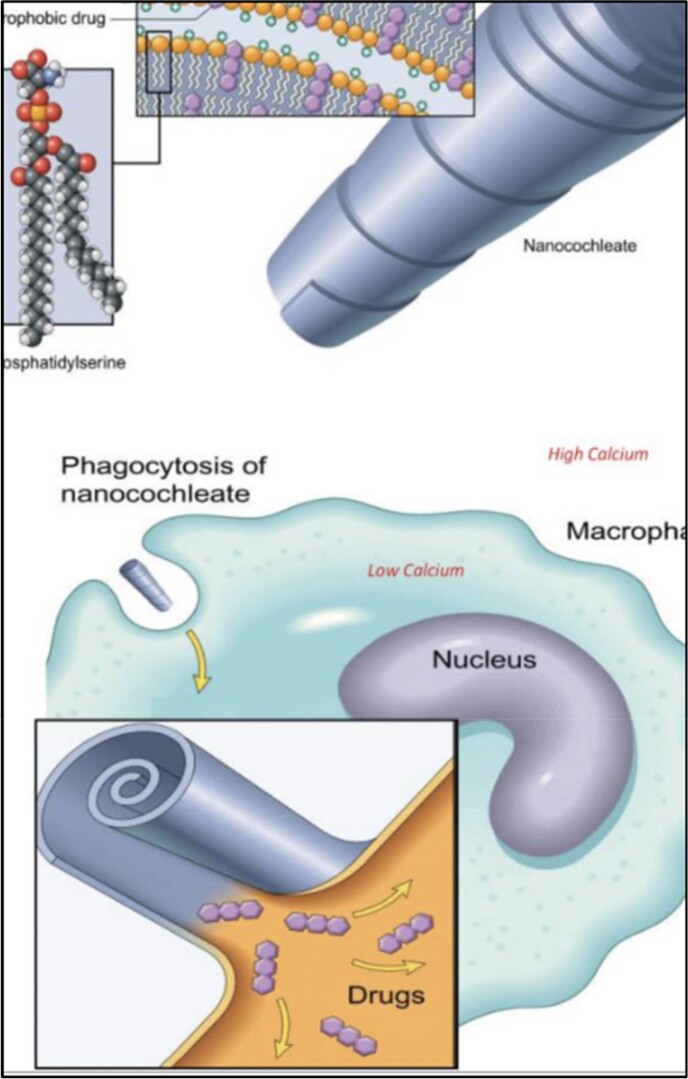

Lipid nanocrystal particle encapsulates amphotericin B molecule and delivers the drug to antigen presenting cells.

**Methods:**

The trial enrolled subjects with HIV who were diagnosed with cryptococcal meningitis and randomized to receive 5-FC and either standard IV amphotericin or LNC amphotericin. Subjects had a lumbar puncture (LP) performed on screening and days 3, 7, and 14. We used broth microdilution methods, standardized by the European Committee on Antimicrobial Susceptibility Testing (EUCAST) to assess the MIC of isolates in each arm of the trial. We tested cryptococcal isolates from the cerebrospinal fluid (CSF) on the day of screening and then on the last positive CSF to contain cryptococcal growth. Statistical analysis included chi-square to test for a difference in MIC between the control and LNC amphotericin groups; and linear regression to compare MIC with early fungicidal activity (EFA), which is a surrogate endpoint.

Intervention and Control Gropus for EnACT Phase 2

**Figure:**
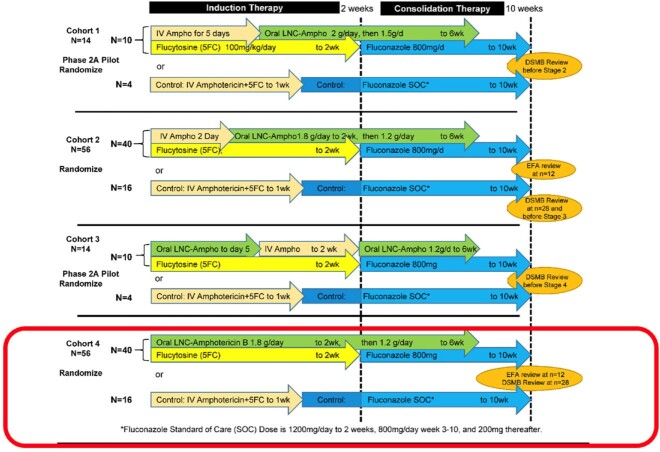

The EnACT trial phase 2 was completed in 4 cohorts to determine the ideal combination of IV and LNC amphotericin. The MIC analysis was completed in the 4th cohort. In the 4th cohort, participants randomized to the intervention group started LNC amphotericin B from the day of enrollment.

Broth Microdilution for 5-Flucytosine Susceptibility Testing

**Figure:**
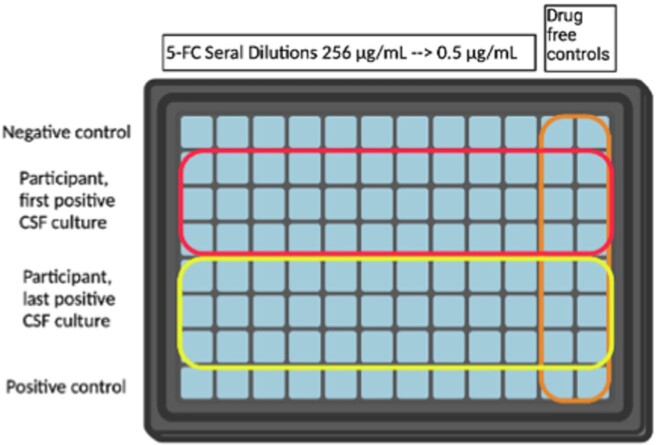

A 96-well plate was prepared with serial dilutions of 5-FC ranging from 256 µg/mL to 0.5 µg/mL in columns 1-10. Columns 11 and 12 were drug-free controls. The first and last CSF isolate from each unique participant was inoculated into rows B-D and E-G, respectively. The first row is a negative control and the last row is a positive control.

**Results:**

The MIC_50_ was 4 µg/mL and MIC_90_ was 8 µg/mL for both the control and LNC amphotericin B groups. There was no evidence of 5-FC resistance in any *Cryptococcus* isolate tested after 2 weeks of therapy. 73% (n=8) of participants in the control group and 73% (n=19) in the LNC amphotericin group had no change or a decrease in MIC from the first to last *Cryptococcus* isolate. 27% (n=3) in the control group and 19% (n=5) in the LNC amphotericin group had a 2-fold increase in MIC. 9% (n=2) in the LNC amphotericin group had a 4-fold increase in MIC. There was no association with MIC and EFA. 5-FC CSF levels were above the MIC_50_.

Distribution of MIC for flucytosine

**Figure:**
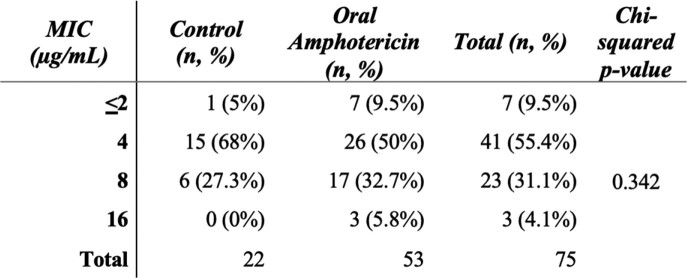

The MIC for each CSF isolate in the control and oral amphotericin B arms of the trial with the percents of each MIC level in each trial arm. Chi-squared statistic testing for a difference in MIC between each trial arm.

Early Fungicidal Activity Compared to MIC for Each Trial Arm

**Figure:**
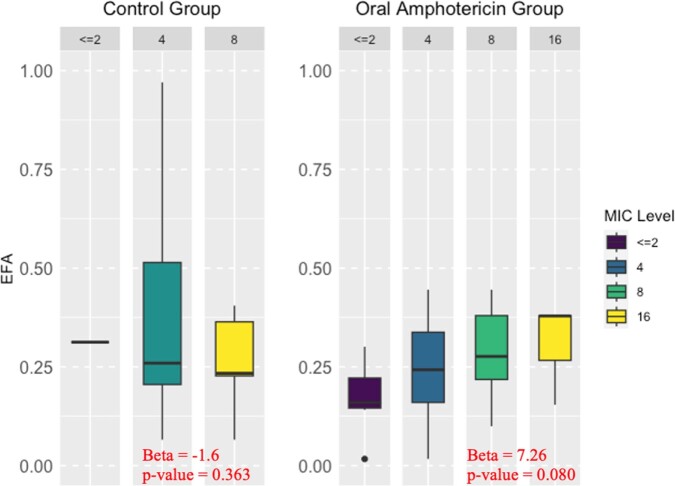

The EFA is compared to MIC in the control and oral amphotericin groups. Linear regression comparing EFA and control group shows that there is no correlation between MIC and EFA.

Change in MIC During Treatment with Flucytosine and Amphotericin B

**Figure:**
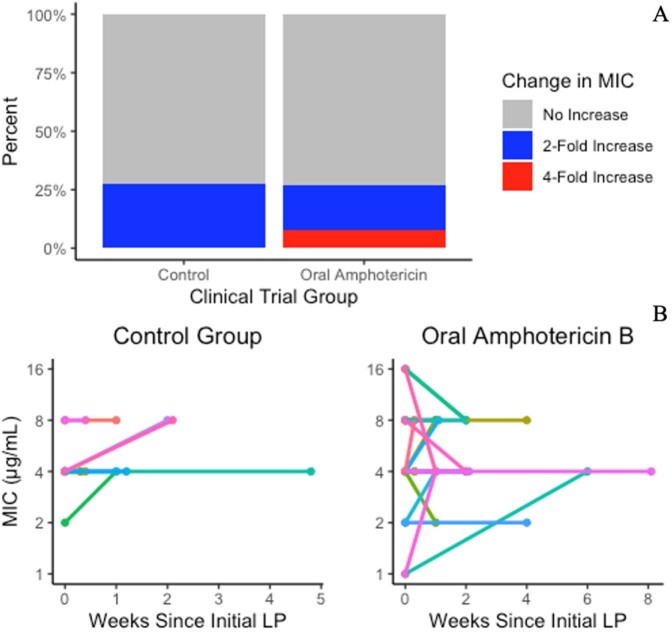

A) Percent of participants in each trial group who had an increase in MIC over the treatment course. B) Individual participants are represented by a line with the MIC represented by a dot on the end of each line.

**Conclusion:**

There is no evidence of baseline resistance to 5-FC or incident resistance during therapy in individuals presenting with cryptococcal meningitis in Uganda. LNC amphotericin B can safely be used in combination with 5-FC.

**Disclosures:**

**All Authors**: No reported disclosures

